# Therapeutic Alliance in COVID-19 Era Remote Psychotherapy Delivered to Physically Ill Patients With Disturbed Body Image

**DOI:** 10.3389/fpsyg.2021.638274

**Published:** 2021-03-24

**Authors:** Nicola Grignoli, Paola Arnaboldi, Mattia Antonini

**Affiliations:** ^1^Servizio di Psichiatria e Psicologia Medica, Organizzazione Sociopsichiatrica Cantonale, Savosa, Switzerland; ^2^Lega Ticinese Contro il Cancro, Bellinzona, Switzerland; ^3^Servizio Medico Psicologico, Organizzazione Sociopsichiatrica Cantonale, Bellinzona, Switzerland

**Keywords:** therapeutic alliance, supportive-expressive psychodynamic psychotherapy, remote psychotherapy, consultation-liaison (C-L) psychiatry, mentalization and reflective function, obesity, cancer, COVID-19

## Abstract

The COVID-19 pandemic outbreak has led to a general reorganization of health services and an increase in outpatient telemedicine in mental healthcare for physically ill people. Current literature highlights facilitators and obstacles concerning the use of new technologies in psychotherapy, an underrated topic of research in the context of supportive expressive psychotherapy. More insight is needed to explore the characteristics of video in therapeutic alliance for treatment of specific mental disorders experienced in psychosomatics, particularly with people suffering from a disturbed body- and self-image. Using two clinical vignettes, it is the authors’ intention to enrich the critical debate on current knowledge in psychosomatic remote psychotherapy, with special focus on mentalization deficits and their impact on therapeutic alliance in the consultation-liaison psychiatry setting. In particular, we will question the interpersonal processes at stake related to mirroring and the disruption caused by the use of videoconference applications. We will also reflect upon the relationship between the therapeutic alliance and the medical team, and that between patient and psychotherapist. The aim is to improve psychotherapeutic alliance maintained during the pandemic for specific mental disorders and to inform about possible clinical factors that could be the subject of future empirical studies or professional guidelines.

## Introduction

The COVID-19 pandemic outbreak has led to a general reorganization of health services and an increase in outpatient telemedicine (Gilli, 2020). Psychological and psychotherapeutic activities were reduced to the minimum with the lockdown of psychiatric outpatient clinics and psychologists’ consulting rooms, which remained accessible only for emergencies. Ordinary mental health treatment was reduced or carried out remotely, in line with the specialist recommendations on mental health interventions during the pandemic (Wright and Caudill, 2020).

Before the COVID-19 pandemic, few studies investigated the efficacy of implementing video conferencing systems in the field of psychotherapy. Remote telepsychotherapy has been demonstrated to be efficacious across different psychiatric disorders, in particular in underserved populations whether with adults, children or the elderly (Bashshur et al., 2016). A work by Norwood et al. (2018) through two meta-analyses showed that working alliance in videoconferencing psychotherapy was inferior to face-to-face delivery but that target symptom reduction was not inferior. COVID-19 era literature highlights facilitators and obstacles concerning telemedicine for mental health, and the debate is ongoing regarding the use of new technologies in psychotherapy during pandemics (Bucci et al., 2019). Professional guidelines available specify the conditions to be met for good practice of teleconsultations in the psychological field: choice of software and IT aspects, privacy, optimization of “telepresence,” economic aspects, emotional factors and need for feedback (The British Psychological Society, 2020). Videoconferencing-based psychotherapy in the psychoanalytic field is more recent and investigations in this field into the specific psychopathology that requires treatment are rare (Juhos and Mészáros, 2019). As yet, there is no literature that empirically investigates thoroughly the characteristics of video in the treatment of specific mental disorders. In videoconferencing, the clinical encounter is reduced to its visual dimension narrowed down to those elements that the medium can transmit. In this sense, the cases of patients suffering from a disturbed body image deserve to be investigated with particular attention.

Body image is an intriguing topic that includes neurological, psychological and sociocultural elements. It has been a longstanding subject of inquiry since the beginning of the twentieth century and has been progressively covered within a large area of investigation in the clinical field (Cash and Smolal, 2012). Disturbed body image (also called distorted or negative body image) is a condition of increased attention, preoccupation and dissatisfaction with one’s shape and weight. When the preoccupations with appearance causing clinically significative distress are accompanied by repetitive behaviors and lack of insight, a diagnosis of body dysmorphic disorder (BDD) could be made following criteria fixed by the DSM-V. Eating disorder, where the preoccupations are mostly focused on weight gain or loss, is a differential diagnosis for BDD but suffering from both conditions is not uncommon (Ruffolo et al., 2006). Eating disorders share many similarities with BDD (Hartmann et al., 2013) and delusional thoughts in particular are investigated in order to include targeted interventions (Konstantakopoulos et al., 2012). To a certain extent, it is possible to understand some psychosomatic disorders, such as eating disorders, as expressions of a deficit in the reflexive function, where the very possibility of constructing and processing mental representations is partially impaired (Skårderud, 2007).

Using two clinical vignettes, from consultation-liaison (C-L) psychiatry activity during COVID-19, it is our intention to discuss current knowledge in psychosomatic videoconferencing psychotherapy, with special focus on emotional state resonance and its impact on therapeutic alliance. In particular, we will discuss the interpersonal processes at stake related to affect mirroring and their disruption with the use of videoconference applications: image splitting in self-view and other-view; risk of emergence of dissociation; risk of emergence of pre-mentalizing modes of thinking. The aim is to question the automatic indication for video psychotherapy during the COVID-19 pandemic and to inform about possible clinical factors that could be the subject of future empirical studies.

## Clinical Vignettes

### Clinical Vignette 1

The C-L psychiatry service received a psychological consultation request from the general practitioner of a 38-year-old woman affected by hepatitis B and suffering from work-related stress condition associated with pathological obesity. She needed psychological support because her weight gain was worsening, and she was suffering from a generalized sense of shame.

During the assessment phase, which took place several years ago, it emerged that the patient had suffered from binge-eating disorders since early adolescence. In the context of migration and traumatic separation, she has progressively developed major obesity with a current BMI of 43.9 (pathological obesity class III) associated with an anxiety-depressive syndrome on avoidant personality traits. For the treatment of the eating disorder and the associated psychic problem, she was initially given psychological support with integrated psychopharmacological treatment and for two years she has been followed with weekly supportive-expressive psychodynamic psychotherapy. The psychological support in the first instance focused on her negative self-image and body image that revealed a dissociation of identity, which then became the core of psychotherapy. We worked on the integration of her self-image and on the deep sense of shame and inadequacy she felt. Re-enacted in her social life (insecure job, economic difficulties) and relational difficulties (living alone), these factors destabilized her psychic state by increasing her dissociation (see alien self), leading to compulsive feeding in order to calm herself. Over the last few years, she has been evaluated several times for bariatric surgery, initially excluded for psychic instability and poor adherence to the necessary food compliance. At the time of writing, weight loss and better food hygiene can be observed, and she is again on the list for gastric bypass surgery.

The regular weekly setting was disrupted by the physical distancing rules introduced during the COVID-19 pandemic at the beginning of March 2020 and, following institutional recommendations, the patient was offered remote psychotherapy. At the first telephone contact, the modalities were discussed and the possibility of using videoconferencing was proposed. This the patient refused because she would be faced with her own image. Specifically, she was able to express and explain to the therapist how the characteristics of video therapy would increase her internal tension. The critical point identified later by the patient turned out to be the confrontation with her own body image on the screen during the video call (self-view). It was thus agreed to continue the therapy by telephone. Thanks to this redefinition of the setting, in which the relationship was maintained through the audio channel only, the patient was able to propose subjects that until then had been difficult to access: affectivity in love relationships.

### Clinical Vignette 2

The C-L psychiatry service received a psycho-oncological consultation request from the haemato-oncologist for a 54-year-old woman recently diagnosed with chronic lymphocytic leukemia. She needed psychological support because of feeling distressed by her gain in weight following corticosteroid during chemotherapy. During the assessment phase, which took place immediately before the COVID-19 lockdown in March 2020, it emerged that the patient had suffered from a lifelong binge-eating disorder. She reached a BMI of 44.1 (pathological obesity class III) in her thirties, when she and her husband divorced after 10 years of marriage. She suffered from panic attack disorder and received benzodiazepine pharmacotherapy but had not at that point received any psychotherapeutic treatment.

From the psychological assessment, it emerged that she had an insecure attachment style with a negative self-image and a negative image of the other. She did not consider herself deserving of help and this was a constant characteristic of her relational life. She agreed to see a psychologist only because the medical oncologist recommended it. She agreed to work through cognitive behavioral techniques as part of cognitive analytic therapy in the psycho-oncological setting in order to cope with the effects of corticosteroids on her food consumption. When the lockdown necessitated the interruption of the face-to-face sessions, she agreed to remote online consultations. During the first teletherapy session, she experienced strong discomfort and asked to interrupt the session stating that she would call later if she needed further help.

The following week, the psychotherapist called the patient to enquire about her emotional state. The patient thanked the therapist for the call and explained that she had found the video session extremely distressing: she had experienced something very similar to the panic attacks she had suffered from in her youth. She found it overwhelming to be confronted with her own image on the screen during the video call. She also explained that hearing the calming voice of the therapist, and simultaneously seeing the therapist’s face on the video was distressing: she felt that she could only concentrate on a single sensory channel at a time. They agreed to continue the therapy by telephone. The patient was followed throughout the chemotherapeutic regimen and was able to deal with her weight increase, seeing it as a side effect of the medical therapy.

## Discussion

### Consultation-Liaison Setting and “the Therapeutic Alliance Triangle”

Requests for psychological interventions in the C-L psychiatry setting are mainly related to problems of compliance and adherence to medical treatment (De Giorgio et al., 2015). This aspect is related to a perspective of studying the therapeutic alliance as a multifaceted construct. Adherence and compliance with medical treatment inform us of the therapeutic alliance between patient and medical team, and between psychologists and psychiatrists and the medical team, since mental health professionals form an integral part of the multidisciplinary team entrusted with resolving such problems (Pai and McGrady, 2014; Arnaboldi et al., 2020).

Once inside the psychotherapist’s consulting room, another therapeutic alliance originates and should be fostered and nurtured: the one between patient and psychotherapist. Here one explores psychological issues other than those related to strictly medical problems such as clinical pathways, bariatric surgery or oncological treatment. In both our clinical vignettes, the risk of disrupting the alliance between the patient and the psychotherapist becomes, from the psychotherapist’s point of view, a threat to the alliance with the medical team. In these complex care settings, a triangle takes shape and faithfully represents the relationships between the patient, who lies at the centre of the process of care, the medical team, whose prime focus is on physical illness goals, and the mental health care professionals. Psychotherapists should maintain a balance between the medical team’s requests and the patient’s needs. As regards the alliance between psychotherapists and patients, the time element is universally recognized as one of the most significant factors (Norcross and Lambert, 2011). In one of our clinical vignettes, the psychological intervention had been ongoing for almost two years and the therapeutic alliance was strong to the point that the patient felt safe enough to disclose her discomfort to the psychotherapist. Thus, it was possible to continue the psychological work, focusing both on issues concerning the patient’s psychopathology and the medical goal of achieving bariatric surgery.

In the second vignette, the psychological intervention had only just started. The patient decided to interrupt the sessions and it was the therapist who decided to renew contact the following week in order to enquire about her discomfort during the video session. This clinical passage offered the patient and the psychotherapist the chance to continue working on the issue of adherence to the medical treatment, pursuing the patient’s objective of survival. This can be considered a specific characteristic of psychological interventions in consultation liaison psychiatry: the psychologist should always be attentive to the influence and interdependence of the alliance with the medical team and the alliance with the patient, balancing the two (Lipowski, 1986) and pursuing an integrative supportive-expressive psychotherapeutic approach (Arnaboldi et al., 2017).

Our clinical vignettes also show the importance of the impact on the therapeutic alliance of strong communication skills, empathy, openness and a paucity of hostile interactions (Lipowski, 1986). In both the vignettes, the psychotherapist was able to mirror and be receptive to the patients’ difficulties, to be open to them and work through alternatives, thereby preserving the therapeutic alliance.

### Remote Consultation as a Threat to the Therapeutic Alliance

In the context of C-L psychiatry, remote counseling offers the considerable advantage of facilitating access to treatment and avoiding the usual architectural and institutional barriers (Hilty et al., 2006). Especially for people with disabilities, a possible physical limit of access to mental healthcare is overcome by technology. However, for some patients who are undergoing psychosomatic consultations, and specifically for people suffering from a disturbed self-image (Ryum et al., 2015), remote consultation focusing on visual representation may prove to be a psychological barrier and a threat to therapeutic alliance.

The refusal to accept the videoconference indicates a crucial clinical problem. Video therapy for patients with eating disorders with negative body image or dissociative symptoms is complicated by self-reflection processes which could be specific to them (Dimaggio and Lysaker, 2018). On the one hand, affect mirroring through the body is in play. The therapist’s face in video maintains a potential for authentic emotional attunement and is an invitation to find and understand one another better. Nevertheless, the whole body is not represented in the frame, which limits the possibility of expressing full attunement. On the other hand, the box that reproduces one’s reflected image conveys (like a mirror) a dissociative potential, which our patient was able to recognize. For the patient, the double mirroring imposed by default by the most commonly used applications could be a source of distress.

The invitation to videoconference, proposed by default during the reorganization of some mental health services caught up in the outbreak of the COVID-19 pandemic, therefore deserves careful evaluation, taking into account the psychopathological characteristics of each clinical case. Similar considerations about emotional dysregulation can be generalized to other areas of social and work-related functioning in which the pandemic has been responsible for a shift from a physical to a distance relationship.

### Faced With Self-Image Through Mirroring

The patient’s psychosomatic disorder can be understood in terms of a deficit of self-regulatory abilities and distortion in mentalization (Dimaggio and Lysaker, 2018). The eating disorder can therefore be seen as characterized by a dissociative process (Gleaves and Eberenz, 1995) aimed at managing emotional tensions triggered by a negative body image (Rosen, 2004).

The empirical study of mind development recognizes the crucial importance of mirroring activity in development processes (Stern, 2010). The reflective function emerges in childhood through adequate interpersonal experiences with the caregiver (Winnicott, 1967). Facial mimicry, words, the rhythm of the caregiver’s activities, all reflect the emotional states of the baby, introducing a subtle but crucial multimodal variation. This mirroring, which must be characterized by coherence, contingency and marking, constitutes a primary form of representation that the caregiver refers to the baby through non-adhesive empathic movement. The repetition of experiences of attuned mirroring within a relationship of secure attachment, are at the origin of the development of the reflective self and the capacity for self-regulation. On the contrary, repeated failed experiences of mirroring, in excess or in defect with respect to the emotional states of the baby, are deposited in the implicit memory, constituting dissociated parts of one’s own identity (alien self, according to the proposed terminology) (Bateman and Fonagy, 2010).

Referring to the theoretical corpus of mentalization, we can therefore state that in the course of face-to-face psychotherapy, confrontation with the responding face of the therapist allows the patient to understand their own emotional activation. Mirroring promotes the regulation of arousal levels and induces positive feelings of containment and emotional contact in the patient (Fonagy et al., 2018). In the context of more severe disorders, particularly in the borderline area, it is possible to hypothesize a distortion of normal early interpersonal mirroring processes. This distortion can have profound effects on the construction of a nuclear unitary sense of the self, emotional self-regulation and the development of mentalization skills (Bateman and Fonagy, 2010). In serious personality disorders, and in general in any clinical situation where the triad composed by sense of self, mentalization and affective regulation is disturbed, an explicit and corrective mirroring activity is crucial. Through mimicry, tone of voice and choice of words, tuned to the patient’s affective state, the therapist promotes a fuller and more authentic contact with the patient’s inner self and a more extended capacity to mentalize (Kramer and Pascual-Leone, 2018).

### Define the Remote Setting for Patients With Self-Image Disturbance

The patients described in the clinical vignettes who preferred a traditional telephone follow-up expressed a refusal to their psychotherapist: the refusal to present themselves in a relational form where the image is central (activating a painful confrontation with their self-image), and so becoming lost in the distressing experience of being mirrored for long minutes in the self-view window.

In a remote psychological consultation, besides the problematic nature of the therapist appearing in close-up (also conveying possible implications and unwanted connotations such as newsreader, police interrogation, video-intercom), there is the addition of the self-view dimension that the most used platforms offer by default in a smaller box next or close to the image of the interlocutor. In the video call, the self-view introduces a mirror effect, where the patient’s expressiveness is constantly reflected. For some people, the fatal attraction of this small mirror is irresistible and disorienting. The facial expression loses its spontaneity and appears accentuated, relational contact loses quality. In this context, mentalization can regress toward less evolved forms, such as “pretend mode”. This expression refers to one of the three forms of pre-mentalistic thinking identified in the context of clinical work with patients suffering from severe personality disorders. The “pretend mode”, a normal part of early development, can be understood in the psychotherapy of personality disorders as an anti-elaboratory discourse. In a “pretend mode,” a patient could talk about their mental state without considering the actual body experience in the here and now of the session (Bateman and Fonagy, 2010). During a conversation using normal applications, the medium implicitly invites one to approach different levels of experience. These levels of experience have already been crossed in part in the course of our psychological development when we looked for ourselves and found ourselves in the face of the attuned other.

The offer of remote therapy with video must be made and handled with caution. Technical details such as the extension of the frame and the possibility of activating or masking the view of oneself, as well as the question of backgrounds (digitized or real), should be made explicit and worked through with the patient at each stage of therapy. If these setting details are not acknowledged and discussed, the treatment itself can slip into an “as if” form, in which patients may talk about mental states but lose contact with genuinely embodied emotional experiences. The medium’s unavoidable dissociative potential risks silently destroying the emotional attuning that is so laboriously sought after. When the patient’s capacity for self-regulation of mental arousal and mentalization has diminished, the audio channel alone is likely to limit excessive stimulation and encourage psychotherapeutic work.

## Conclusion

Our main task as psychotherapists in medical settings is twofold: to strive to obtain adherence to medical treatment as requested by the medical team; and to deal with the psychological functioning of the patient by pursuing the psychotherapeutic alliance. These two aspects are often interdependent.

As regards the patient’s psychological functioning, the psychotherapist should promote the integration of the self and emotional regulation, and accompany patients toward a wider capacity for thought. Our responsibility is to create and protect a physical and relational context in which these crucial abilities can be restored or developed. It is not certain that video therapy always possesses this quality. At this point, we can reflect on the well-known work by Peter Fonagy and restate his question, “What Works For Whom?” (Roth and Fonagy, 2005). Where the pathology itself is structured around the alteration of self and body image, the dissociative effects are amplified. In any event, the complexity introduced by video seems excessive. The single voice channel of the phone call perhaps possesses interesting qualities and is not necessarily outdated as an opportunity to take us beyond the current health crisis.

## Data Availability Statement

The original contributions presented in the study are included in the article/supplementary material, further inquiries can be directed to the corresponding author/s.

## Ethics Statement

Written informed consent was obtained from the individual(s) for the publication of any potentially identifiable images or data included in this article.

## Author Contributions

NG and PA redacted the clinical vignettes. MA mainly developed the theoretical perspective. All authors contributed equally to writing and reviewing the text and conceived article perspective.

## Conflict of Interest

The authors declare that the research was conducted in the absence of any commercial or financial relationships that could be construed as a potential conflict of interest.
